# Next-generation nanomaterials for environmental remediation: smart design, hybrid materials and sustainable use

**DOI:** 10.3389/fchem.2026.1772161

**Published:** 2026-03-18

**Authors:** Hina Singh, A. S Dhanu, Abhayraj S. Joshi, Ivan Mijakovic, Priyanka Singh

**Affiliations:** 1 Division of Biomedical Sciences, School of Medicine, University of California, Riverside, CA, United States; 2 Department of Biotherapeutics Research, Manipal Academy of Higher Education, Manipal, India; 3 Systems and Synthetic Biology Division, Department of Life Sciences, Chalmers University of Technology, Gothenburg, Sweden; 4 The Novo Nordisk Foundation, Biotechnology Research Institute for the Green Transition, Technical University of Denmark, Kongens, Lyngby, Denmark; 5 Department of Health Technology, Section of Experimental and Translational Immunology, Technical University of Denmark, Kongens, Lyngby, Denmark

**Keywords:** AI-driven material design, environmental remediation, hybrid nanomaterials, life cycle assessment, microplastics, PFAS, sustainability, green synthesis

## Abstract

Environmental contamination has increased steadily over recent decades due to industrialization, urban expansion, intensive agriculture, and improper waste management. As a result, a wide range of pollutants, including per- and polyfluoroalkyl substances (PFAS), microplastics, pharmaceutical residues, endocrine-disrupting compounds, and heavy metals are now frequently detected in water, soil, and sediment systems worldwide. Many of these contaminants are chemically stable, persist for long periods in the environment, and can accumulate in living organisms, posing significant toxicological and ecological risks and making their removal particularly challenging. Engineered nanomaterials have emerged as promising tools for pollutant removal because of their tunable surface chemistry, and ability to interact with contaminants through multiple mechanisms. This review examines recent advances in eco-engineered nanomaterials for environmental remediation, with particular attention to green strategies, major material classes and their underlying removal mechanisms. Across the studies discussed, adsorption-based and hybrid systems frequently report high removal efficiencies for metals and dyes under controlled conditions, while framework-based materials show improved selectivity toward persistent pollutants (including PFAS) through combined electrostatic, hydrophobic, and hydrogen-bonding interactions. Photocatalytic and redox-active systems are highlighted for accelerating the degradation of recalcitrant organics through reactive oxygen species–mediated pathways. Recoverable designs, including magnetic and scaffold-immobilized composites, are also emphasized because they are often reported to retain substantial performance over multiple reuse cycles. Sustainability and deployment challenges are also discussed, including life-cycle assessment, material reuse, environmental fate, toxicity risks, and data-driven strategies for design and optimization.

## Graphical Abstract

**Figure:**
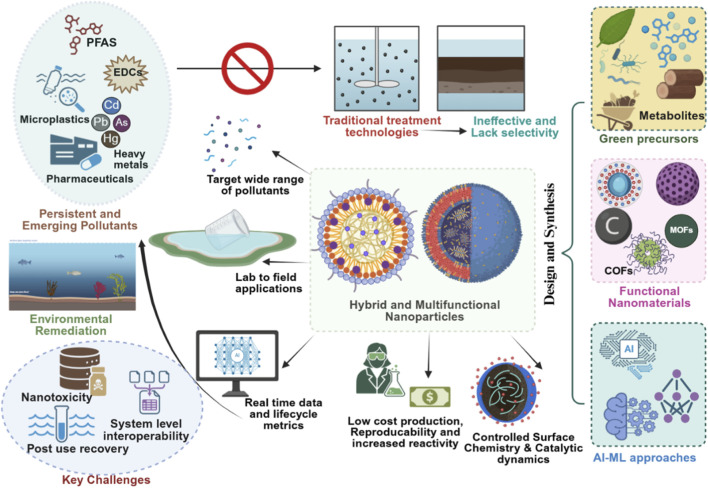

## 1 Introduction

The escalating pace of anthropogenic activity across industrial, agricultural, and urban sectors has led to a sustained increase in the release of chemically diverse pollutants into both terrestrial and aquatic ecosystems (Hou et al., 2025). Among these, persistent and emerging contaminants such as per- and polyfluoroalkyl substances (PFAS), micro- and nanoplastics, pharmaceuticals, endocrine-disrupting compounds (EDCs), and extracellular genetic materials have been detected with growing frequency in surface water and groundwater worldwide (Chang et al., 2026; Ullah et al., 2022). These pollutants are often characterized by high chemical stability, long environmental persistence, bioaccumulative potential, and complex toxicological behavior, which makes their degradation or removal using conventional technologies particularly challenging (Srivastava and Malik, 2026; Wang et al., 2024). Importantly, growing evidence from environmental toxicology studies indicates that chronic exposure to low concentrations of such contaminants, particularly in complex mixtures, can induce sub-lethal effects, endocrine disruption, antimicrobial resistance selection, and long-term ecosystem-level disturbances. Conventional remediation methods, including coagulation, membrane filtration, and granular activated carbon adsorption, offer foundational treatment capabilities but are frequently inadequate when faced with chemically resilient, low-molecular-weight, or nanoscale contaminants. Moreover, these approaches often result in phase transfer rather than true detoxification, leading to contaminant accumulation in secondary waste streams such as sludge, where remobilization and trophic transfer remain significant concerns (Rath et al., 2025). In addition, these technologies often lack selectivity, regenerative capacity, and consistent performance in complex contaminant matrices where chemical and biological hazards coexist. Such limitations underscore the urgent need for advanced remediation strategies capable of addressing both the chemical diversity of emerging contaminants and the dynamic conditions of real environmental systems (Ahmad et al., 2023; Giao et al., 2017). Recent investigations into heavy metal toxicity, environmental fate modeling, and advanced wastewater treatment frameworks emphasize that remediation strategies must be evaluated not only by removal efficiency, but also by transformation pathways, residual toxicity, and long-term environmental risk reduction (Lee Y. H. et al., 2025). (Dong et al., 2025; Um et al., 2025).

Engineered nanomaterials (ENMs) represent a promising alternative remediation approach due to their high surface-area-to-volume ratios, tunable surface chemistries, and intrinsic catalytic and antimicrobial properties (Bandala and Berli, 2019)^,^ (Takallu et al., 2024). These characteristics enable ENMs to operate through multiple synergistic mechanisms, including adsorption, redox-mediated transformation, and microbial inactivation, offering a versatile platform for contaminant-specific and multifunctional pollutant removal. Recent studies indicate that ENMs can function as effective adsorbents for plastic particles**,** with removal mechanisms involving electrostatic attraction, pore-filling effects, and hydrophobic interactions, enabling separation of microplastic and nanoplastic fractions from complex water matrices. Similarly, inorganic nanomaterials, including metal oxides, zero-valent metals, and nanocomposites, have been widely investigated for the broad-spectrum removal of water contaminants such as dyes, heavy metals, oils, pharmaceuticals, and plastic-associated pollutants. From a wastewater treatment perspective, these materials are increasingly evaluated within multi-barrier treatment trains, where adsorption, catalytic degradation, and post-treatment recovery are combined to minimize secondary pollution and operational trade-offs. In parallel, sustainability considerations, including raw material sourcing, life-cycle leaching behavior, reuse potential, and regeneration efficiency, are increasingly prioritized in the design of these nanomaterials**.** However, the practical deployment of ENMs is strongly influenced by how these materials are synthesized, as fabrication routes directly affect surface chemistry, stability, toxicity, and environmental compatibility.

In recent years, green synthesis methods that employ plant extracts (Singh and Mijakovic, 2022; Singh and Mijakovic, 2024), microbial metabolites (Sing et al., 2022; Singh et al., 2022; Singh and Mijakovic, 2025), or agro-waste-derived precursors reduce dependence on toxic reagents, organic solvents, and energy-intensive processes (Madhusudanan et al., 2025a; Singh et al., 2016a), while simultaneously introducing biologically derived surface functionalities that can enhance colloidal stability, pollutant affinity, and biocompatibility in complex environmental matrices. Importantly, many of these routes rely on low-cost, widely available feedstocks and relatively mild processing conditions, making them more compatible with scalable and reproducible production compared to conventional chemical synthesis.

The integration of computational modeling and data-driven approaches is increasingly influencing the design and optimization of ENMs. Machine learning (ML) algorithms informed by physicochemical descriptors and structure–activity relationships enable high-throughput screening of material–pollutant compatibility and preliminary evaluation of environmental safety profiles (Kamal et al., 2025). Moreover, the combination of artificial intelligence (AI) with real-time sensing technologies is enabling the development of adaptive remediation systems capable of responding to fluctuating environmental conditions. Such intelligent systems are particularly relevant for wastewater treatment applications, where influent composition, contaminant loading, and toxicity profiles vary temporally and spatially. When integrated with biological and conventional treatment units, nanotechnology-based wastewater treatment systems can evolve into smart, ENM-enabled processes that support improved system-level efficiency and scalable waste management. In this review, the term “smart” nanomaterials refers to systems that extend beyond passive adsorption and exhibit adaptive or responsive functionality under environmental conditions. This includes stimulus-responsive behavior (e.g., pH-, redox-, light-, or magnetic-field-triggered activity), dynamically tunable surface chemistry that enhances contaminant selectivity, and materials whose design or deployment is supported by data-driven optimization. Such smart nanomaterials are intended to adjust their reactivity, selectivity, or recoverability in response to changing environmental matrices, thereby improving performance and long-term sustainability.

Nevertheless, critical knowledge gaps remain, particularly regarding the environmental fate, bioaccumulation behavior, and ecotoxicity of ENMs and their transformation products (Varg and Svanbäck, 2023). Studies on metal- and sulfide-based nanosystems indicate that aging, sulfidation, and redox cycling can significantly alter particle reactivity and ion release, thereby influencing both remediation efficiency and ecological risk. The absence of standardized testing protocols and harmonized regulatory frameworks continues to limit large-scale implementation and risk assessment. Addressing these challenges requires an integrated evaluation of material performance, environmental safety, and deployment feasibility. Accordingly, the present review examines recent advances in eco-engineered nanomaterials, with particular emphasis on hybrid material architecture, sustainable synthesis pathways, contaminant-specific targeting strategies, and intelligent deployment concepts.

## 2 Eco-engineered nanomaterials

Eco-engineered nanomaterials represent an advancement beyond conventional pollutant treatment technologies to multifunctional, life-cycle-conscious solutions. These materials are not merely high-performing at the nanoscale; they are designed with sustainability, adaptability, and long-term environmental compatibility in mind (Guidi et al., 2023). Their value lies not only in pollutant removal but also in their potential to align with global green chemistry principles, reduce lifecycle emissions, and operate effectively across diverse environmental conditions. In environmental remediation, eco-engineered nanomaterials most commonly include metallic and metal oxide nanoparticles (e.g., Fe, Ag, TiO_2_, CeO_2_), carbon-based materials (e.g., biochar, graphene derivatives), and porous frameworks (MOFs and COFs), which function through adsorption, redox transformation, photocatalysis, or antimicrobial mechanisms. The effectiveness and environmental safety of these materials are strongly governed not only by their composition, but also by their surface chemistry, stability, and interaction with natural matrices, properties that are directly shaped by the synthesis route.

**FIGURE 1:**
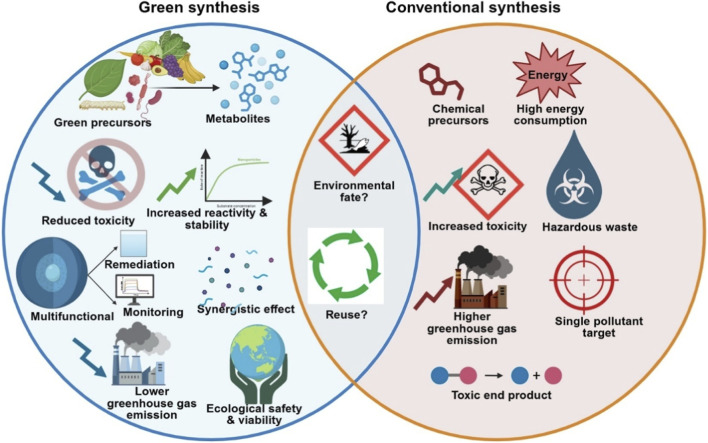
Comparison of green and conventional nanomaterial synthesis routes for environmental remediation, highlighting differences in precursors, toxicity, energy demand, multifunctionality, and environmental impact.

As a result, the concept of eco-engineering inherently links material functionality with how nanomaterials are produced, deployed, and recovered in environmental systems. A central innovation driving this shift is the refinement of green synthesis protocols (Singh et al., 2025). While traditional nanoparticle fabrication often involves toxic reductants and energy-intensive processes, the recent decade has seen an explosion of bio-assisted methods leveraging plant extracts, microbial metabolites, and agro-waste derivatives (Figure 1) (Singh et al., 2016a; Singh et al., 2018a). These green synthesis routes are particularly relevant for environmental remediation because they produce nanomaterials with biologically derived surface functionalities that influence adsorption affinity, redox behavior, colloidal stability, and ecotoxicity in water and soil systems. Such surface modifications frequently improve contaminant binding and reduce unintended toxicological effects. (Singh et al., 2018b; Yuan et al., 2024). From a scalability perspective, bio-assisted synthesis also offers improved batch-to-batch reproducibility when standardized biomass sources or reusable extracts are employed, while reducing overall material and energy costs. For instance, biosynthesized iron or silver nanoparticles (AgNPs) have demonstrated comparable or superior reactivity to their chemically produced counterparts while exhibiting better colloidal stability in variable pH or ionic strength conditions (Conde-Cid et al., 2021). One notable advancement is the use of lignin, a naturally abundant biopolymer, as both a reducing and stabilizing agent for synthesizing AgNPs. Lee et al. (2020) developed lignin-mediated AgNPs via pulsed laser irradiation and ultrasonication, yielding particles with a diameter of ∼7–8 nm that exhibited high catalytic efficacy in reducing toxic nitroaromatic compounds (e.g., 4-nitrophenol, nitrobenzene) and demonstrated selective sensing capabilities for Hg^2+^ and H_2_O_2_ (Lee et al., 2021). This dual function, pollutant degradation and biosensing, illustrates the multifunctional potential of green-synthesized nanomaterials for both remediation and monitoring applications. Bio-assisted remediation studies, including Cd(II) biosorption using bamboo stem–derived biochar and microbial removal of Cr(VI) by *Shewanella putrefaciens* MTCC 8104, further demonstrate the effectiveness of biomass- and microbe-based strategies for heavy metal removal from contaminated wastewater (Sable et al., 2024; Sable et al., 2025).

Beyond the method of synthesis, the defining strength of eco-engineered nanomaterials is their ability to participate in multiple pathways for pollutant transformation (Giese et al., 2020). Their functional roles extend beyond adsorption and include redox-mediated breakdown, catalytic conversion, and even pathogen neutralization. Nanostructured oxides such as CeO_2_ and MnOx can cycle between oxidation states, facilitating degradation of phenolics, EDCs, and nitroaromatics *via* reactive oxygen species (ROS). Similarly, doped photocatalysts such as visible-light-active TiO_2_ exhibit high quantum yields in transforming recalcitrant organic pollutants without the need for harsh oxidants or UV energy input. The synergy between surface chemistry, catalytic dynamics, and environmental responsiveness enables these materials to perform effectively in mixed-contaminant matrices, a scenario where conventional technologies often fail (Ma et al., 2018).

A significant development in this field is the shift toward composite and hybrid systems, where multiple nanoscale mechanisms are integrated into a single architecture (Xiong et al., 2022). By embedding redox-active particles onto adsorptive scaffolds such as biochar, graphene oxide (GO), or porous silicates, researchers have engineered materials that simultaneously immobilize heavy metals, catalyze organic degradation, and resist fouling (Zhang X. et al., 2025). These hybrid systems show promise in treating complex pollution scenarios such as wastewater containing both microbial pathogens and antibiotics, where multifunctionality is not optional but essential (Venkateshbabu et al., 2024). The metal-oxide and carbon-based nanomaterials have been shown to possess efficient adsorption and removal capabilities for heavy metals from the water. At the same time, they are easier to regenerate and cause less secondary pollution than the conventional adsorbents (Hussein et al., 2025).

While green synthesis and hybrid architecture have introduced important advances, the true potential of eco-engineered nanomaterials lies in how these design strategies influence their physicochemical behavior in real-world systems. Their ability to respond to environmental cues, mediate selective interactions, and operate across diverse contaminant profiles continues to expand as more nuanced material configurations are explored. Understanding these relationships at the material level is essential for advancing both performance and safety in complex remediation scenarios. Critically, this new generation of nanomaterials is being evaluated not just for removal efficiency but also for ecological safety and economic viability over repeated use cycles (Amaya-Santos et al., 2025). Recent life cycle assessment (LCA) models have quantified the greenhouse gas emissions, water footprint, and toxicological burden associated with nanomaterial production, providing a data-rich foundation for safe-by-design principles (Komaei et al., 2025; Langhorst et al., 2025). Together, these advances in green synthesis, hybrid architectures, and life-cycle-aware design establish the foundational principles of eco-engineered nanomaterials. Importantly, these strategies align closely with circular economic principles, where waste-derived precursors are valorized into functional nanomaterials, and material recovery, regeneration, and reuse are integrated into the design phase rather than treated as post-deployment considerations. By coupling pollutant removal with feedstock recycling and multi-cycle material utilization, eco-engineered nanomaterials shift remediation frameworks from linear consumption models toward resource-circulating systems.

## 3 Functional nanomaterials for environmental interfaces

**FIGURE 2:**
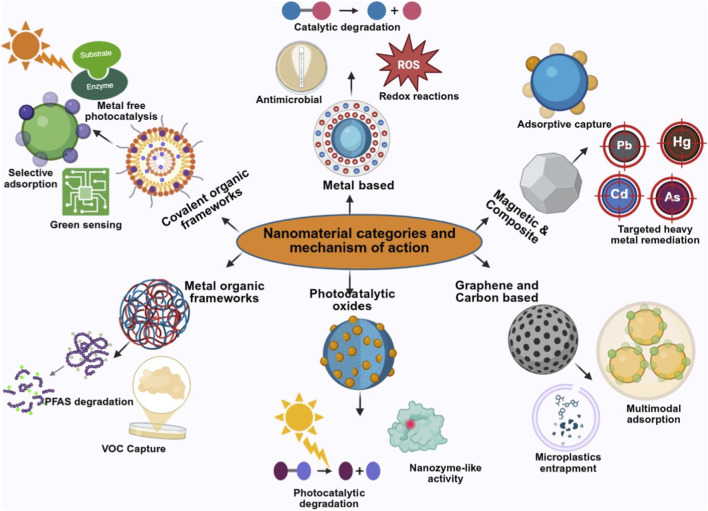
Major classes of nanomaterials and their dominant mechanisms in environmental remediation, including adsorption, catalytic and photocatalytic degradation, redox reactions, antimicrobial activity, sensing, and microplastic entrapment.

While eco-engineered nanomaterials share common sustainability-driven design principles, their remediation performance is ultimately dictated by composition, structural architecture, and surface functionality at environmental interfaces. Recent advances have moved beyond generic adsorption toward materials capable of redox transformations, photocatalysis, and selective ion capture under realistic conditions (Berkani et al., 2022). In this section, we examine key classes of nanomaterials, including metals, oxides, carbon-based structures, and frameworks, not simply by their composition but by the distinct mechanistic pathways they leverage for pollutant degradation, selectivity, and regeneration under complex environmental matrices (Figure 2; Table 1).

**TABLE 1:** Risk–benefit comparison of eco-engineered nanomaterials for environmental remediation.

Nanomaterial	Primary function	Target pollutants	Green synthesis feasible	Main challenges
AgNPs	Antimicrobial, catalytic degradation, ROS generation	Pathogens, dyes, antibiotics, endocrine disruptors	Plant, fungal, microbial extracts; food/agro-waste	Ion leaching (Ag^+^), aggregation, resistance development, cost
FeNPs (nZVI)	Reductive dechlorination, Fenton-like oxidation, metal sequestration	Cr(VI), Pb(II), TCE, nitrate, chlorinated hydrocarbons	Biomass leachate, compost, phytosynthesis	Surface passivation, short lifespan, air/soil instability
MNPs (Fe_3_O_4_)	Magnetic recovery, adsorptive capture, carrier for hybrids	Cd(II), As(III), oil residues, pharmaceuticals	Partially-biogenic Fe precursors, templated iron oxides	Oxidative degradation, magnetic loss, synthesis complexity
Graphene/CNTs	Multimodal adsorption, hybrid support, microplastic entrapment	PAHs, heavy metals, antibiotics, micro/nanoplastics	Biochar, hydrothermal carbon, algal residues	Aggregation, toxicity at high doses, environmental persistence
TiO_2_/ZnO	Photocatalytic degradation, nanozyme-like activity, antimicrobial coatings	Dyes, pesticides, EDCs, pharmaceuticals, biofilms	Bio-precursor mediated synthesis, fungal templating, visible-light doping	Solar activation efficiency, particle aggregation, dopant leaching, ROS-related toxicity
CeO_2_ NPs	Redox catalysis, enzyme mimicry, oxidative stress mitigation	Phenols, bisphenols, VOCs, EDCs, antibiotics	Limited-some microbial and plant-derived methods	Incomplete environmental fate data, redox cycling uncertainties
MOFs	Modular adsorption, catalysis, PFAS degradation, VOC capture, MOF-based hybrids	Arsenic, fluoride, PFAS, CO_2_, antibiotics, pesticides, EDCs	Mechanochemical, ligand-free, biotemplated synthesis emerging	Hydrolytic instability, high cost, fine particle recovery, ecotoxicity data gaps
COFs	Metal-free photocatalysis, selective adsorption, green sensing	Bisphenol A, MB, NOx, VOCs, H_2_O_2_ production, sulfur compounds	Organic base catalysis, ligand functionalization from green routes	Low long-term durability in aqueous systems, crystallinity control, scalability

### 3.1 Metal-based nanomaterials

Previous assessments of nanotechnology for contaminated site cleanup highlight the broad application of metal, metal oxide, carbon, and composite nanomaterials, particularly for the removal of heavy metals, dyes, and emerging pollutants from wastewater and groundwater systems. These studies increasingly emphasize sustainability metrics, including life-cycle emissions, metal ion release, and material reusability, reinforcing the shift toward eco-engineered nanomaterials designed for sustainable remediation rather than single-use pollutant removal. Among the most widely studied are silver and iron nanoparticles, which offer distinct antimicrobial and redox capabilities, respectively (Ashrafi et al., 2022). Green AgNPs exhibit high efficacy in inactivating a broad spectrum of bacteria, viruses, and fungi (Singh et al., 2016b; Singh et al., 2018a). Their mode of action, driven by ROS generation and membrane disruption, has proven useful in co-remediation scenarios where chemical and microbial pollutants coexist (Doan et al., 2020). AgNPs also serve as plasmonic photocatalysts under visible light, enabling the degradation of dyes, antibiotics, and EDCs (Devi et al., 2022). For instance, AgNPs synthesized using *Sargassum ilicifolium* demonstrated vigorous bactericidal activity and effectively degraded malachite green and methylene blue dyes in aqueous media, achieving up to 100% removal under light exposure (Devi et al., 2022). However, despite their effectiveness, challenges such as uncontrolled Ag^+^ ion release, cytotoxicity in non-target organisms, and environmental persistence under acidic conditions limit their long-term deployment. In particular, emerging evidence indicates that AgNPs can be taken up and accumulated by terrestrial and aquatic organisms, potentially triggering toxic responses that vary by species and exposure context (Tortella et al., 2020). These concerns underscore the need for improved understanding of uptake pathways, tolerance mechanisms, and ecotoxicological behavior under environmentally realistic conditions. Strategies such as matrix encapsulation, surface passivation, and bimetallic hybridization have been employed to modulate their leaching behavior and enhance recovery.

In contrast, iron-based nanoparticles, particularly zero-valent iron and iron oxides, have been widely investigated for their redox reactivity and comparatively lower environmental risk. Notably, Plachtová et al. synthesized superparamagnetic iron oxide nanoparticles using green tea extract, converting initially toxic GTFe precursors into more stable, eco-friendly particles. These green FeNPs achieved 93% degradation of malachite green dye at 125 mg Fe/L within 60 min, while exhibiting no toxicity in most tested organisms up to 1 g/L (Plachtova et al., 2018). Rónavári et al. demonstrated a scalable approach by reusing green waste residues, such as green tea, coffee arabica, and *Virginia creeper*, for the multi-round synthesis of FeNPs. Notably, FeNPs synthesized from three successive green tea extractions retained high activity, degrading chlorinated VOCs with efficiencies of 91.0%, 83.2%, and 68.5%, respectively (Ronavari et al., 2023). This highlights the feasibility of using agrowaste-derived extracts and multi-cycle biomass utilization for environmental remediation.

While green Ag and Fe-based nanomaterials exhibit improved stability and reduced toxicity, multi-laboratory evidence from Bondarenko et al. (2016) highlights the inherent ecotoxicological risks associated with metal ion leaching, with *Daphnia magna* and *R. subcapitata* serving as sensitive indicators (Bondarenko et al., 2016). On the other hand, Cerium oxide nanoparticles (CeO_2_), or nanoceria, have been investigated for their unique redox-switching ability between Ce^3+^ and Ce^4+^ states. This feature enables both oxidative degradation of persistent organic pollutants (POPs) and ROS scavenging in stressed aquatic environments (Kusmierek, 2020). Recent studies have demonstrated near-complete degradation of phenolic compounds using CeO_2_-based nanocatalysts over multiple regeneration cycles without loss of activity. Beyond chemical remediation, nanoceria has shown potential in mitigating oxidative stress in wetland systems by buffering excess ROS, thus protecting native microbial populations (Putri et al., 2025). Nonetheless, the long-term bioavailability and transformation behavior of CeO_2_ in environmental matrices remain poorly understood.

### 3.2 Magnetic and composite nanoparticles

Magnetic nanoparticles (MNPs), particularly those based on magnetite (Fe_3_O_4_) and maghemite (γ-Fe_2_O_3_), are of growing interest due to their retrievability and functional tunability (Madhusudanan et al., 2025b). Their magnetic properties enable post-treatment separation, significantly improving the reuse potential and minimizing the release of residuals into ecosystems (Paul et al., 2023; Singh H. et al., 2018). Functionalization of MNPs with amine, thiol, or carboxyl groups has expanded their application range to include heavy metals such as arsenic, cadmium, and mercury, as well as organic pollutants and oil residues (Farrukh et al., 2013). For example, Farrukh et al. developed polymer-brush-grafted Fe_3_O_4_ nanoparticles functionalized with dithiocarbamate (DTC) groups, achieving complete removal of Hg^2+^ ions from water. Compared to monolayer analogs, these MNPs exhibited higher chelating group density, superior adsorption capacity, and faster kinetics, demonstrating the power of surface engineering for targeted heavy metal remediation (Farrukh et al., 2013). In oil spill contexts, hydrophobic MNPs have been used to selectively absorb hydrocarbons, allowing rapid magnetic recovery with minimal energy input.

Despite their advantages, MNPs are sensitive to environmental conditions, as high salinity, extreme pH, and oxidative stress can degrade their magnetic cores and functional coatings, thereby limiting long-term stability and reuse (Khalid et al., 2022). Encapsulation in polymer matrices or covalent grafting to inert scaffolds has been proposed as a solution, though such modifications often increase synthesis complexity and cost. Balancing surface activity with environmental resilience remains an ongoing design challenge (Khalid et al., 2022).

### 3.3 Graphene and carbon-based nanomaterials

Carbon-based nanomaterials, especially GO, reduced graphene oxide (rGO), and carbon nanotubes (CNTs), offer an exceptional platform for multifunctional pollutant remediation. Their π–π conjugation, surface functionalization potential, and chemical stability allow them to interact with a wide array of contaminants through hydrophobic interactions, electrostatic attraction, and complexation. For example, GO nanosheets have demonstrated a high sorption capacity for antibiotics, dyes, pesticides, and metal ions. In contrast, rGO, with its higher conductivity and reduced oxygen content, excels in electron transfer-based catalysis (Hu et al., 2019). Importantly, many carbon nanomaterials can now be synthesized from agricultural residues, including coconut shells, coffee grounds, and sugarcane bagasse, aligning with circular economy models (Balusamy et al., 2023).

Biochar, a mesoporous carbonaceous material derived from biomass pyrolysis, has been widely employed for the adsorption of heavy metals, pharmaceuticals, and PFAS precursors (Zhang X. et al., 2025). Surface modification using oxidants or metal nanoparticles further enhances adsorption selectivity and catalytic reactivity. These biosorption and bioremediation studies demonstrate well-defined mechanisms, including surface complexation between functional groups and metal ions, redox transformation of toxic species (e.g., Cr(VI) to Cr(III)), and biologically mediated uptake when carbon-based supports are integrated with microbial or enzymatic systems**.** Together, these features position biochar-based and biohybrid platforms as effective components of hybrid bio-nano remediation frameworks.

Beyond dissolved chemical contaminants, the porous architecture and tunable surface chemistry of carbon-based materials also enable interactions with particulate pollutants. Microplastics and nanoplastics are now recognized as among the most abundant and widely distributed environmental pollutants. Recent studies highlight the ability of carbon-based adsorbents, including biochar and carbon aerogels, to capture and immobilize plastic particles through surface adsorption and physical entrapment mechanisms, offering a viable strategy for mitigating plastic pollution in aquatic systems (Munzel et al., 2025; Nihart et al., 2025).

However, despite their promise, GO-based materials raise unresolved questions regarding their bio interface behavior, particularly under variable ionic conditions in aquatic environments. Recent findings by Jing et al. (2022) show that *Pseudomonas aeruginosa* PAO1 exhibits nonlinear attachment behavior on GO surfaces, with maximal binding observed at moderate ionic strengths (200–400 mM NaCl) due to enhanced contact area and reduced electrostatic repulsion. Conversely, low (1–100 mM) and high (600 mM) ionic strengths reduce bacterial adhesion due to electrostatic repulsion and the collapse of the polymer layer, respectively (Jing et al., 2022). These insights suggest that ionic strength plays a crucial role in dictating the antibiofouling performance of GO. This factor must be considered in real-world deployments involving saline, wastewater, or matrices with fluctuating pH levels. The long-term fate of CNTs, in particular, raises concern due to their structural persistence and potential cytotoxicity, especially under chronic exposure scenarios.

### 3.4 Oxide-based photocatalytic nanomaterials (TiO2, ZnO, and related systems)

Semiconductor oxides such as titanium dioxide (TiO_2_) and zinc oxide (ZnO) remain foundational in photocatalytic water treatment due to their ability to generate ROS under UV or visible light. Despite their broad utility, challenges like wide band gaps and rapid electron-hole recombination limit their effectiveness under ambient conditions. To enhance performance, strategies such as doping, hybridization, and heterojunction design have been employed to address these intrinsic limitations. For instance, Fe_2_O_3_/ZnO core–shell heterostructures demonstrated superior Rhodamine B degradation due to synergistic band alignment and improved charge separation (Wu et al., 2012). Similarly, ZnO/CdS heterostructures have shown enhanced visible-light-driven photocatalysis, leveraging bandgap engineering and controlled morphology to outperform their individual components (Nadikatla et al., 2023). Integration of nanomaterial-based photocatalysis (metal oxides, composite oxides) with traditional or biological methods for wastewater treatment could not only provide a strong multi-barrier solution but also enhance the deterioration of organic substances and, at the same time, lessen the usage of strong chemicals or very low/high pH conditions. Emerging alternatives, such as bismuth-based photocatalysts (e.g., BiVO_4_, Bi_2_WO_6_), offer narrow bandgaps and strong visible light absorption, effectively degrading dyes and antibiotics (Oladipo and Mustafa, 2023). However, issues like nanoparticle aggregation, photocorrosion, and environmental persistence remain.

### 3.5 Metal-organic frameworks

Metal–organic frameworks (MOFs) are highly porous crystalline materials composed of metal ions or clusters coordinated with organic ligands. Their defining features, extremely high surface area, tunable pore size, and structural versatility make MOFs exceptional adsorbents for a wide range of environmental contaminants (Rasheed, 2023). Recent advances have led to the design of MOFs capable of selectively capturing heavy metals, dyes, antibiotics, and even greenhouse gases such as CO_2_ from air and water systems. One of the most promising applications of MOFs is in carbon capture and sequestration (CCS). Their high surface area and customizable pore chemistry enable efficient CO_2_ uptake even under humid conditions, which are common in industrial emissions (Rego et al., 2022). In a 2023 report, a zinc-based MOF demonstrated outstanding CO_2_ adsorption capacity at low partial pressures, showcasing its potential in flue gas purification. In the context of water purification, MOFs functionalized with specific groups (e.g., amines, thiols, carboxyls) have been employed to remove trace metals such as arsenic, lead, and chromium (Zhu et al., 2022). MOFs have also been investigated for the capture and degradation of pharmaceutical compounds, with enzyme-mimetic or catalytic MOFs providing added functionality for pollutant breakdown. More recently, MOFs have been explored for the selective removal of radionuclides such as uranium, cesium, and strontium from wastewater, offering high-capacity alternatives to conventional remediation techniques (Sheta et al., 2023).

Despite these advantages, MOFs face several challenges that limit their practical deployment. Many MOFs are susceptible to hydrolytic degradation, which compromises their structural integrity in aqueous environments (Bahrani-Pour et al., 2025). Research is now focused on engineering water-stable MOFs, particularly zirconium- and aluminum-based frameworks for improved durability under environmental conditions. Additionally, scalability and cost of synthesis remain significant hurdles. Green synthesis routes using plant-derived ligands or solvent-free mechanochemical approaches are under active investigation to reduce costs and environmental impact. Moreover, integrating MOFs into composite systems such as MOF-polymer membranes, MOF-graphene hybrids, or MOF-magnetic particle composites can enhance mechanical robustness, enable targeted pollutant capture, and facilitate post-treatment recovery. Recent innovations include the incorporation of dual-active-site MOFs such as Co-MOF-74-SH into nanofiltration membranes for selective selenium (Se) removal. Zhang et al. (2025) reported that Co-MOF-74-SH-modified membranes achieved up to 99.6% Se removal, outperforming bare membranes (∼74.4%) through a synergistic mechanism of adsorption and salt-salt separation. DFT simulations confirmed strong binding between SeO_3_^2-^ and sulfur sites, while the membrane also maintained high water permeance and divalent/monovalent salt selectivity (>80) (Zhang H. et al., 2025). Similarly, recent work by Bahrani-Pour et al. introduced two 3D water-stable silver-sulfur MOFs (SCU-1 and SCU-2), which demonstrated exceptional iodine uptake capacities (3.650 and 3.749 g/g, respectively) and high H_2_S adsorption energy, setting a new benchmark for capturing volatile contaminants in humid environments (Bahrani-Pour et al., 2025). These results highlight the potential of MOF-based membranes and frameworks for precision ion removal, gas adsorption, and water resource recovery in complex aqueous environments. Such hybrid systems hold great promises for real-time sensing, selective adsorption, and catalytic remediation in both water and air purification platforms.

### 3.6 Covalent Organic Frameworks

Covalent Organic Frameworks (COFs) are crystalline, porous materials composed entirely of light elements (C, H, O, N, B) linked *via* strong covalent bonds into two- or three-dimensional architectures. Their modular design, tunable porosity, and chemical stability make them ideal platforms for environmental applications, especially where metal-free, sustainable materials are desired. Unlike MOFs, COFs offer intrinsic advantages such as lower toxicity and enhanced framework flexibility for functionalization. Recent studies have demonstrated their potential not only in pollutant adsorption but also in photocatalytic processes driven by visible light. For example, Sánchez-Naya and Beuerle developed a β-ketoenamine-linked covalent organic framework (BDP-TFP-COF) incorporating BODIPY photosensitizer units directly into the COF backbone. This metal-free framework exhibited high mesoporosity, broad visible-light absorption, and remarkable multifunctionality, efficiently removing Bisphenol A and methylene blue from water, enabling fluorescence-based sensing, and performing visible-light-driven oxidation of a mustard gas simulant (Sanchez-Naya and Beuerle, 2025). Zhou et al. (2024) enhanced the crystallinity of TpBD-COFs by switching from acetic acid to organic base catalysts, yielding a 2.1-fold improvement in photocatalytic hydrogen peroxide production from air and water under ambient conditions without any sacrificial agents (Zhou et al., 2025). This green, metal-free route to H_2_O_2_ generation highlights how rational COF design can couple environmental remediation with clean energy generation in practical water systems.

While numerous studies report high removal efficiencies under controlled laboratory conditions, it is important to note that many of these systems exhibit performance decline when exposed to realistic environmental matrices containing natural organic matter, competing ions, fluctuating pH, and variable redox conditions. Common failure modes include nanoparticle aggregation, surface passivation, ion leaching, photocorrosion, structural collapse of porous frameworks, and reduced regeneration efficiency over repeated cycles. In several cases, removal is achieved through phase transfer rather than complete mineralization, raising concerns about secondary waste management and long-term environmental burden. These limitations underscore the need for standardized benchmarking under field-relevant conditions rather than reliance on optimized batch experiments alone.

## 4 Hybrid and multifunctional platforms

**FIGURE 3:**
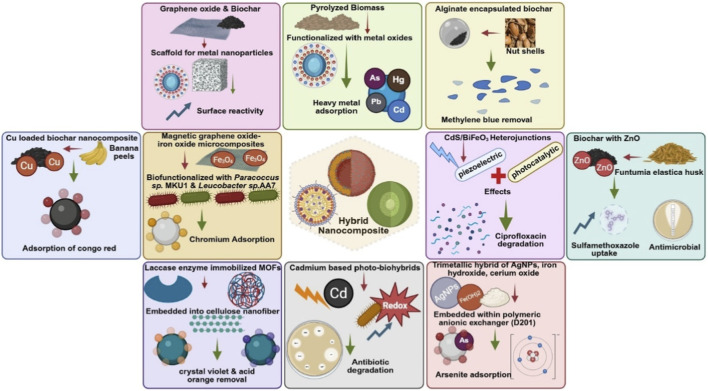
Representative hybrid nanocomposites and their dominant mechanisms for environmental remediation, including adsorption, catalytic degradation, redox transformation, antimicrobial activity, and magnetic recovery across diverse pollutant classes.

The inherent limitations of individual nanomaterials, such as rapid aggregation, narrow pollutant specificity, or degradation under environmental conditions, have driven the emergence of hybrid platforms that integrate multiple nanoscale components into cohesive, multifunctional architectures (Dube et al., 2025). These systems are no longer conceived as additive combinations of active materials; instead, they represent deliberate, synergistic constructs engineered to simultaneously harness adsorption, catalytic degradation, redox cycling, and, in some cases, contaminant-specific sensing (Figure 3) (Bandeira et al., 2025). The convergence of these functionalities within a single platform has enabled unprecedented versatility in treating diverse and co-occurring pollutants in complex aqueous matrices. For example, carbonaceous substrates such as graphene oxide and biochar have been widely employed as scaffolds for metal or metal oxide nanoparticles, offering improvements in dispersion, surface reactivity, and interfacial electron transport (Xiong et al., 2022). Synthesized from pyrolyzed biomass and further functionalized with metal oxides, these hybrid materials have demonstrated dual functionality: effective adsorption of heavy metals and catalytic degradation of organic pollutants (Essa et al., 2024). Their compatibility with circular economy principles, low synthesis cost, and scalability make them particularly attractive for field applications, especially in resource-constrained regions. Essa et al. (2024) exemplified this approach by developing a CuO-loaded biochar nanocomposite synthesized from banana peels (CuO_0_._5_/BC_0_._5_). The material exhibited a high adsorption capacity of 233.6 mg/g for Congo red dye, conforming to Langmuir and pseudo-second-order kinetic models, and demonstrated a spontaneous, endothermic sorption process. This study not only underscores the value of integrating catalytic metal oxides with biochar but also illustrates the effectiveness of waste-derived supports in tailoring nanomaterial functionality (Essa et al., 2024). Similarly, Bahsaine et al. (2024) developed alginate-encapsulated biochar beads from argan nutshells (BC/Alg) for methylene blue removal. These composite beads maintained their structural integrity over multiple cycles, achieving 96.4% dye removal initially and retaining over 82% efficiency after four regeneration cycles. The success of the alginate-biochar coupling lifespan underscores their potential in enhancing recyclability (Bahsaine et al., 2024). Additional multifunctionality was demonstrated by Amaku et al. (2024), who modified *Funtumia elastica* husk-derived biochar with ZnO nanoparticles to yield FBZC nanocomposites. These materials achieved a sulfamethoxazole uptake capacity of 59.3 mg/g and exhibited notable antimicrobial activity, demonstrating dual remediation capacity against pharmaceutical and microbial pollutants. Kinetic studies confirmed chemisorption-dominated mechanisms, while morphological analyses revealed an enhancement in porosity and reactive surface area upon ZnO integration. Such results reinforce the utility of metal–biochar nanohybrids for targeting pollutants with disparate physicochemical properties (Amaku and Mtunzi, 2024).

Further innovation is emerging from biohybrid systems that integrate biological elements, such as enzymes, microorganisms, or biodegradable polymers, into nanostructured frameworks (Sourgi and Dehnavi, 2025). These responsive materials not only sequester pollutants but also catalyze their transformation into less toxic or inert byproducts by mimicking natural metabolic and redox processes (Le and Wang, 2025). A notable example is the multifunctional composite developed by Sourgi and Dehnavi (2025), in which laccase enzymes were immobilized within MIL-100 MOFs and embedded in quaternized cellulose nanofibers. This system achieved greater than 95% removal of both cationic (crystal violet) and anionic (acid orange 7) dyes, underscoring the synergistic effect of enzyme activity and adsorptive scaffold engineering (Sourgi and Dehnavi, 2025). Similarly, Aravind et al. (2022) developed magnetic GO–iron oxide microcomposites biofunctionalized with *Paracoccus* sp. MKU1 and *Leucobacter* sp. AA7 for Cr(VI) bioremediation in a 10 L *ex situ* bioreactor. These platforms achieved high adsorption (up to 272.6 mg/g) and complete reduction to Cr(III), alongside greater than 88% recovery *via* electromagnetic separation and greater than 90% desorption efficiency, demonstrating the feasibility of large-scale bio-nano integration for heavy metal detoxification (Aravind et al., 2022). Further, CdS-based photo-biohybrids have demonstrated efficient antibiotic degradation by leveraging bacterial redox processes under low-intensity light, offering resilience in variable water chemistries (Song et al., 2025). In parallel, chitosan-based hydrogels, though not strictly biohybrids, are advancing as versatile green adsorbents. Their tunable porosity, strong affinity for dyes and heavy metals, and ability to be crosslinked using eco-friendly chemistries make them promising candidates for sustainable remediation (Yang et al., 2025). Recent developments in hydrogel mechanical integrity and multi-cycle usability further expand their potential for deployment in real-world aqueous systems (El-Araby et al., 2023).

Building on the evolution of hybrid nanostructures, a particularly promising avenue lies in electrochemical nanoremediation systems, which couple redox-active nanomaterials with external energy sources such as low-voltage electricity or visible light. These systems incorporate multifunctional components, such as TiO_2_–graphene photoanodes or Fe_3_O_4_-based cathodes, to generate reactive species *in situ* for contaminant breakdown. For example, CdS/BiFeO_3_ step-scheme heterojunctions combine piezoelectric and photocatalytic effects to degrade antibiotic residues, such as ciprofloxacin, achieving up to 9.5 times enhanced removal efficiency compared to conventional photocatalysts. These composites exploit both mechanical and light stimuli to activate singlet oxygen (^1^O_2_) and valence-band holes (h^+^), while demonstrating strong durability and reusability (Yu et al., 2025). In another example, a trimetallic hybrid composed of AgNPs, iron hydroxide, and cerium oxide, embedded within a polymeric anion exchanger (D201), exhibited a high arsenite adsorption capacity (40.12 mg/g). This performance surpassed Ag-free counterparts due to a synergistic triad of adsorption, surface complexation, and redox transformation, while maintaining material stability over repeated cycles (Li et al., 2025). These multifunctional constructs underscore how rational material integration can extend catalytic lifetimes, minimize regenerant use, and target chemically diverse pollutants under ambient or low-energy conditions. Together, the rise of carbon-based hybrids, biohybrids, and electrochemical composites reflects the growing versatility of nanomaterial-enabled remediation. However, their real-world deployment hinges not only on performance but also on durability, environmental safety, and integration into broader treatment systems. Hybrid architecture that enable magnetic separation, scaffold immobilization, or bead encapsulation directly support circular remediation models by facilitating material retrieval, regeneration, and controlled reuse across multiple treatment cycles. Addressing challenges like leaching, fouling, and synthesis scalability requires standardized evaluation and life-cycle-aware design. As these systems advance from lab to field, their potential will be defined by how effectively they target the most persistent and elusive contaminants.

## 5 Addressing emerging contaminants: PFAS, microplastics, and biogenic pollutants

As nanomaterial systems advance, their relevance is most pronounced in addressing emerging contaminants, such as PFAS, microplastics, and biogenic pollutants, which are characterized by extreme persistence, molecular complexity, and resistance to traditional treatment methods. Addressing these contaminants also underscores systemic lack of standardized detection, highlighting gaps in detection methods, limited ecotoxicity data, and regulatory inertia. For example, microplastics act as carriers for endocrine disruptors and antibiotic resistance genes (ARGs), highlighting the need for cross-disciplinary solutions that bridge materials science, environmental microbiology, and policy frameworks (Li et al., 2024).

PFAS removal has achieved success through amine-functionalized MOFs (e.g., UiO-66-NH_2_), which utilize electrostatic and hydrogen-bonding interactions to attain an efficiency of over 90% (Lin et al., 2024). Recent innovations, such as Ti_3_C_2_–UiO-66 hybrids, combine MXene conductivity with MOF porosity, allowing over 94% PFAS removal through a combination of hydrophobic partitioning and catalytic degradation (Ahmadijokani et al., 2023). For microplastics, high-efficiency capture (>85%) has been demonstrated using graphene membranes and magnetic chitosan-CNT composites. However, fouling and fragment release remain challenges. Hybrid systems that incorporate photocatalytic degradation using graphene–metal oxide or MOF-based catalysts are being explored to address both removal and breakdown of plastic residues under ambient conditions.

The meta-analysis of MOF-derived PFAS removal pointed out that selectivity is the result of a combination of electrostatic interactions, hydrophobic partitioning, and hydrogen bonding, and testing realistic water matrices (pH, ionic strength, natural organic matter) was considered crucial to evaluate actual performance in the field. (Lee D. et al., 2025). Complementing material-based strategies, bioremediation using microbial consortia has emerged as a low-impact alternative for degrading polymers like polyethylene and polystyrene (Ihsanullah et al., 2024). These systems, while slower, can be integrated into hybrid platforms, leveraging enzymatic fragmentation with nanostructured supports for more efficient treatment.

A less recognized but critical class includes biogenic pollutants, such as extracellular RNA and microbial DNA, which contribute to the environmental spread of ARGs (Singh et al., 2023). Materials such as cationic iron oxides and functionalized graphene offer selective nucleic acid capture *via* electrostatic interactions, supporting the development of genetic pollution remediation technologies (Singh et al., 2024). Integrating AI-guided material design with pollutant-specific remediation strategies presents a path toward next-generation, adaptive treatment platforms.

## 6 Intelligent and sustainable nanomaterial design

### 6.1 Sustainability metrics and life cycle implications

The transition from high-performing laboratory materials to field-deployable, environmentally safe nanotechnologies demands careful attention to sustainability across the material life cycle. From precursor sourcing to post-use recovery, emerging frameworks in LCA are being used to evaluate nanomaterials not only in terms of their remediation efficacy, but also their environmental trade-offs, synthetic footprint, and waste management implications (Heia et al., 2025).

A growing body of research has highlighted the benefits of green synthesis routes over traditional chemical methods. These greener routes eliminate toxic solvents and minimize hazardous waste generation, while often improving particle dispersion and biocompatibility. The integration of agricultural waste materials as carbon sources or stabilizers further supports the goals of a circular economy.

Similarly, GO derived from biomass such as coconut shells or sugarcane bagasse demonstrates a substantially lower greenhouse gas (GHG) emission profile compared to chemically exfoliated GO (Silva et al., 2024). These alternatives also reduce risks associated with acid treatment waste and occupational hazards. Mechanochemical synthesis of MOFs and is increasingly viewed as an industrially relevant route due to reduced solvent use, lower capital requirements, and improved process scalability. In addition to these scalability benefits, this approach offers sustainability advantages, including improved atom economy and process simplification. These solvent-free methods are especially relevant in decentralized manufacturing or low-resource settings where high-temperature calcination or solvent recovery systems may not be feasible.

Embedding LCA early into material design is now recognized as essential for safe-by-design frameworks (Boyes et al., 2017). Within a circular economy context, LCA also enables quantification of regeneration efficiency, secondary waste minimization, and end-of-life recovery pathways, ensuring that nanomaterial-enabled remediation systems reduce net environmental burden rather than shift impacts across life-cycle stages. When coupled with data on nanotoxicity, reusability, and environmental fate, such assessments enable the prioritization of materials that balance high performance with environmental and occupational safety (Wang and Wu, 2023). This alignment of synthesis optimization with impact modeling will likely play a central role in regulatory evaluation and commercialization.

### 6.2 AI-driven design and smart remediation systems

**FIGURE 4:**
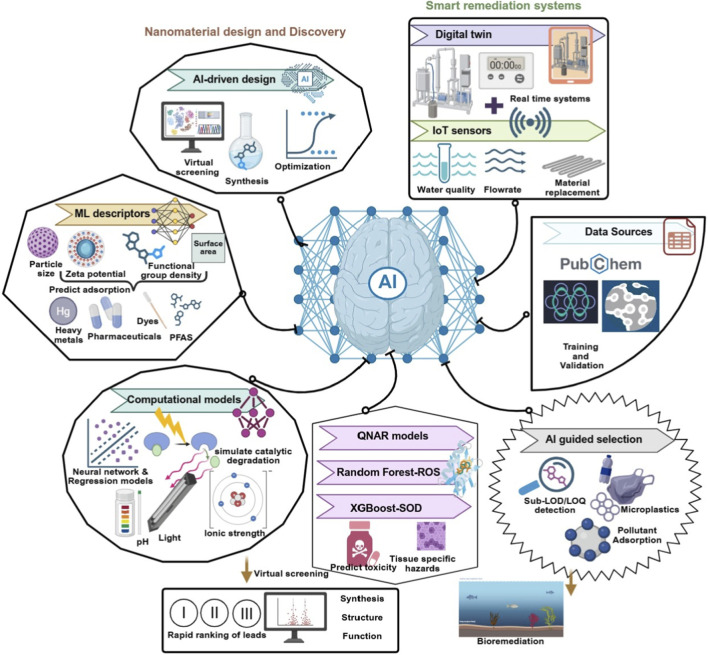
AI-enabled frameworks for nanomaterial design, performance prediction, toxicity assessment, and real-time optimization in smart environmental remediation systems.

Advances in AI and ML are accelerating nearly every phase of nanomaterial development, from virtual screening of precursor combinations to deployment optimization. ML models trained on physicochemical descriptors such as particle size, zeta potential, surface area, and functional group density can now predict adsorption capacities for metals, dyes, pharmaceuticals, and PFAS analogues with increasing precision (Figure 4) (OĞUztÜRk, 2025).

Neural networks and regression models are also being used to simulate catalytic degradation efficiency, particularly in photocatalytic systems under varying pH, ionic strength, and light conditions. These computational frameworks allow for the rapid ranking of candidate materials and the virtual exploration of synthesis–structure–function landscapes. In systems where pollutant interactions are too complex for direct modeling, hybrid approaches that combine statistical learning with empirical validation showing promise in narrowing the experimental scope while capturing key design insights.

Significantly, AI applications now extend to the operational phase of remediation. Digital twins, virtual representations of real-time systems, are being developed to simulate contaminant flux, material saturation, and performance drift under variable conditions (Lakshmi et al., 2021). When coupled with IoT-enabled sensors for water quality monitoring, these systems allow autonomous adjustment of flow rates, regeneration intervals, or material replacement, thereby increasing system resilience and lowering maintenance burdens (Ashkanani et al., 2024).

Beyond performance prediction, AI-driven frameworks are increasingly being applied to address engineering constraints associated with real-world deployment. Data-driven optimization has been used to guide immobilization strategies, including embedding nanomaterials within membranes, polymeric matrices, or packed-bed reactor configurations, in order to minimize particle loss and maintain activity under continuous-flow conditions. Machine-learning-assisted process simulations further enable prediction of pressure drop, material fouling, and efficiency decay over prolonged operation, supporting rational design of regeneration cycles and operational lifetimes.

Data sources powering these tools are expanding rapidly. Open-access platforms such as the Materials Project, NanoCommons, and the PubChem Nanomaterial Repository provide structured datasets for training and validation. Quantitative nanostructure–activity relationship (QNAR) models are also enabling the prediction of ecotoxicity based on surface chemistry, bandgap, and charge density, offering critical early screening capabilities for environmental compatibility. For instance, Wang et al. (2023) applied mechanism-driven machine learning to characterize oxidative stress induced by TiO_2_ nanoparticles in bivalves. Using 36 models across six algorithms and oxidative biomarkers (e.g., ROS, SOD, CAT, GST), they identified exposure concentration, duration, and organ-specific accumulation (notably in the gills and digestive gland) as key drivers of toxicity. Models such as Random Forest–ROS and XGBoost–SOD yielded high predictive accuracy on both training and external validation datasets, establishing a robust framework for predictive nanotoxicology and tissue-specific hazard assessment (Wang et al., 2023). As demonstrated by Noventa et al. (2023), improper treatment of sub-LOD (limit of detection) or sub-LOQ (limit of quantification) data can significantly bias risk assessments, especially in component-based mixture analyses. By integrating such uncertainty modeling into AI-driven systems, remediation decisions can better reflect real-world variability, particularly in trace-level pollutant monitoring for PFAS or pharmaceutical mixtures (Noventa et al., 2024).

In parallel, AI-guided material selection is being extended to emerging challenges such as microplastic capture and additive removal, where models mapping surface interactions between nanomaterials and aged plastics or associated chemicals (e.g., bisphenol A, phthalates) inform the design of multifunctional interfaces capable of both adsorption and catalytic degradation. When combined with lifecycle assessment constraints and toxicity screening, these approaches support the development of remediation systems that are not only adaptive, but also engineered for long-term stability and safe deployment.

### 6.3 Risk–benefit matrix for nanomaterial deployment

As the portfolio of available nanomaterials expands, structured tools are necessary to assess trade-offs between performance and ecological or regulatory risks (Table 2). A “risk–benefit matrix” provides a framework for balancing key parameters such as contaminant removal efficiency, ecotoxicity, material recyclability, and cost-effective scalability (Chen et al., 2024). For example, AgNPs offer strong antimicrobial and catalytic performance. Still, their ion leaching behavior, potential for bioaccumulation, and resistance-induced microbial shifts position them in a high-benefit but high-risk quadrant. In contrast, biochar-supported nanomaterials tend to offer moderate performance with low residual toxicity, placing them in a more favorable zone for low-cost and decentralized systems. Graphene-based materials, while performing well in adsorption, face challenges in recovery and aggregation control, and are thus best deployed where post-treatment separation is feasible. This risk–benefit framing is not static; it evolves as new data emerge from ecotoxicological testing, LCA modeling, and AI prediction pipelines. Embedding such tools into both research and decision-making processes can help prioritize not just what works in the lab, but what will work and persist safely in complex environmental systems.

**TABLE 2:** Comparison of nanomaterials based on remediation efficiency, environmental risk, recyclability, scalability, and overall benefit–risk balance.

Nanomaterial	Removal efficiency	Environmental risk	Recyclability	Scalability	Overall benefit-risk position
AgNPs	High (antimicrobial, dye removal)	Medium–High (Ag^+^ leaching)	Moderate	Moderate	High-risk, high-benefit
Fe_3_O_4_ MNPs	Moderate–High (metals, pharma)	Low–Moderate	High	High	Low-risk, high-benefit
MOFs	High (selective pollutants)	Medium	Low–Moderate	Low–Medium	Medium-risk, high-benefit
Biochar–ZnO	Moderate (pharma + pathogens)	Low	High	High	Low-risk, moderate-benefit
CNTs	High (adsorption)	Moderate–High	Moderate	Low	Medium-risk, moderate-benefit

The “Overall Benefit–Risk Position” represents a qualitative synthesis of multiple performance and safety-related parameters. In this context, “Benefit” considers contaminant removal efficiency, functional versatility, and applicability across pollutant classes, while “Risk” reflects factors such as material toxicity, ion or byproduct leaching, environmental persistence, recovery and regeneration challenges, lifecycle footprint, and synthesis cost.

## 7 Field deployment, barriers, and translational pathways

While laboratory-scale studies underscore the transformative potential of nanomaterials in environmental remediation, real-world deployment is still constrained by multifaceted technical, economic, and regulatory challenges. Overcoming this translational gap requires not only performance optimization but also interdisciplinary co-development, infrastructure readiness, harmonized regulation, and robust field validation.

One persistent hurdle is scalability. High-performing materials like CNTs require energy-intensive synthesis methods such as chemical vapor deposition (CVD). Their colloidal instability under field conditions leads to aggregation and reduced reactivity. While surface functionalization improves dispersion and selectivity, it also introduces complexity, cost, and potential occupational hazards (Zhang et al., 2019).

Equally critical are engineering considerations related to immobilization, continuous-flow operation, and long-term operational stability. Nanomaterials optimized in batch systems often exhibit diminished performance when translated to flow-through reactors due to shear forces, fouling, and material loss. As a result, immobilization strategies, such as integration into membranes, monolithic supports, polymer composites, or magnetic matrices have emerged as essential design elements for maintaining performance while enabling material recovery and reuse.

For *in situ* remediation, nanoscale zero-valent iron (nZVI) remains among the most field-tested nanomaterials. However, its deployment in heterogeneous subsurface environments is constrained by mobility limitations. In a recent numerical and statistical study, Asad et al. (2021) analyzed the transport of carboxymethyl cellulose (CMC)-stabilized nanoscale zero-valent iron (nZVI) in heterogeneous aquifers using a two-dimensional model. Their findings revealed that solution viscosity and injection rate significantly influence travel distance, while groundwater velocity and nZVI concentration had negligible effects. Additionally, subsurface heterogeneity strongly impacted particle dispersion, and short lag periods between injections reduced nZVI attachment losses (Asad et al., 2021). These insights offer critical design parameters to optimize injection strategies and minimize performance loss due to clogging or aggregation in real-world remediation settings.

MNPs, such as Fe_3_O_4_ and γ-Fe_2_O_3_, offer post-treatment retrievability and composite integration; however, they are prone to oxidation and dissolution in acidic or saline matrices. Functional coatings enhance stability but again complicate synthesis and raise costs. Partial recovery of MNPs during regeneration may also disrupt microbial communities and redox equilibria (Dahiya et al., 2025).

Long-term operational stability remains a major bottleneck across nanomaterial platforms, particularly under variable pH, salinity, and contaminant loading. Performance drift due to surface passivation, aggregation, or structural degradation has been reported for metal oxides, MOFs, and carbon-based systems alike. Addressing these challenges requires coupling materials engineering with process-level optimization, where AI-assisted monitoring and control systems enable real-time adjustment of operational parameters such as flow rate, residence time, and regeneration frequency.

Cerium oxide (CeO_2_), with redox-switching capacity, remains effective for persistent organic pollutants. New Fe-CeO_2_ nanozymes have shown degradation of EDCs under ambient conditions. However, their long-term ecological fate and bioaccumulation potential remain poorly characterized, necessitating long-duration studies. MOFs, despite high surface area and tunability, suffer from hydrolytic fragility and mechanical breakdown in aqueous systems. Embedding Zr/Al-based MOFs into membranes or integrating with MXenes or graphene has improved PFAS removal, but introduced lifecycle and cost concerns. Despite growing proof-of-concept success, most nanomaterials remain at the pilot stage, and several reported systems have failed to maintain laboratory-level efficiencies under continuous-flow or field conditions, hindered by fragmented regulation. Regulatory agencies, such as the U.S. EPA and EU REACH, still lack nano-specific guidelines that account for the dynamic behavior, transformation products, and long-term persistence in ecosystems (Gao et al., 2021). Although OECD TG 318 and ISO/TR 19057 offer preliminary protocols for nanoparticle dispersion and basic characterization, they fall short on lifecycle fate, bioaccumulation, and ecosystem-level risk benchmarking.

## 8 Future outlook and concluding perspectives

The rapid evolution of eco-engineered nanomaterials offers significant potential to address the growing complexity of environmental contaminants. As pollution profiles shift toward low-concentration, persistent, and co-occurring threats such as PFAS, pharmaceuticals, microplastics, and EDCs, the demand for next-generation materials that are selective, multifunctional, and resilient continues to increase. Future developments are likely to focus on precision-engineered platforms, such as ligand-gated MOFs, biomimetic nanostructures, and hierarchical composites (e.g., Ag–graphene–Fe hybrids). These materials combine adsorption, catalytic degradation, and antimicrobial properties within responsive architectures that adjust their activity based on environmental triggers, such as pH, redox state, or contaminant concentration, while offering opportunities to minimize secondary pollution through controlled reactivity, recoverability, and multi-cycle reuse.

In parallel, the shift toward green and scalable synthesis is driving more sustainable material production strategies. Strategies such as mechanochemical fabrication, enzyme-assisted assembly, and biomass upcycling (from sources like coconut shells, sugarcane bagasse, and banana peels) significantly reduce the environmental footprint. Coupling these approaches with LCA ensures sustainability is embedded at the design phase, not as an afterthought, and enables early-stage evaluation of toxicity trade-offs, scalability constraints**,** and material aging behavior.

AI and ML are increasingly integrated into material discovery and system optimization. Predictive models based on physicochemical descriptors can forecast material–contaminant interactions and potential transformation pathways relevant to environmental risk assessment. Integration of nanosensing technologies with IoT-linked monitoring platforms supports adaptive treatment systems, particularly in decentralized wastewater scenarios. Emerging attention is also directed toward extracellular genetic pollutants, including viral RNA, plasmids, and ARGs. Functional nanomaterials such as cationic iron oxides and GO conjugates demonstrate nucleic acid binding capacity, though challenges related to disposal, regeneration, and biosafety remain.

Despite these advances, many high-performance materials remain at pilot scale, constrained by regulatory fragmentation, limited standardized testing, and scale-up challenges. Long-term environmental fate, ecotoxicity, and performance stability under realistic conditions require further investigation. Field trials with standardized benchmarking and transparent reporting of both successful outcomes and failure modes are essential to accurately assess technological readiness. Thus, eco-engineered nanomaterials are shifting environmental remediation from passive removal toward systems capable of detection, transformation, and adaptive response. Their successful implementation will depend on balancing performance with environmental safety, regulatory alignment, scalability, and long-term sustainability.
